# A Novel Rapid Test to Detect Anti-SARS-CoV-2 N Protein IgG Based on Shear Horizontal Surface Acoustic Wave (SH-SAW)

**DOI:** 10.3390/diagnostics11101838

**Published:** 2021-10-05

**Authors:** Yu-Chi Peng, Chia-Hsuan Cheng, Hiromi Yatsuda, Szu-Heng Liu, Shih-Jen Liu, Takashi Kogai, Chen-Yen Kuo, Robert Y. L. Wang

**Affiliations:** 1 Biotechnology Industry Master and PhD Program, Chang Gung University, Taoyuan 33302, Taiwan; jasmine170819@gmail.com; 2 Tst Biomedical Electronics Co., Ltd., Taoyuan 324, Taiwan; joshcheng@tst.bio (C.-H.C.); yatsuda.hiromi@tst.bio (H.Y.); justinliu@tst.bio (S.-H.L.); kogai.takashi@tst.bio (T.K.); 3 National Institute of Infectious Diseases and Vaccinology, National Health Research Institutes, Miaoli 35053, Taiwan; levent@nhri.org.tw; 4 Japan Radio Co., Ltd., Saitama 356-8510, Japan; 5 Division of Pediatric Infectious Diseases, Department of Pediatrics, Chang Gung Memorial and Children’s Hospital, Linkou 33305, Taiwan; jennykster@gamil.com; 6 Department of Biomedical Sciences, College of Medicine, Chang Gung University, Taoyuan 33302, Taiwan

**Keywords:** surface acoustic wave (SAW), severe acute respiratory syndrome coronavirus 2 (SARS-CoV-2), in vitro diagnostic (IVD)

## Abstract

Since the Coronavirus disease 2019 (COVID-19) pandemic outbreak, many methods have been used to detect antigens or antibodies to severe acute respiratory syndrome coronavirus 2 (SARS-CoV-2), including viral culture, nucleic acid test, and immunoassay. The shear-horizontal surface acoustic wave (SH-SAW) biosensor is a novel pathogen detection platform with the advantages of high sensitivity and short detection time. The objective of this study is to develop a SH-SAW biosensor to detect the anti-SARS-CoV-2 nucleocapsid antibody. The rabbit sera collected from rabbits on different days after SARS-CoV-2 N protein injection were evaluated by SH-SAW biosensor and enzyme-linked immunosorbent assay (ELISA). The results showed that the SH-SAW biosensor achieved a high correlation coefficient (R = 0.9997) with different concentrations (34.375–1100 ng/mL) of the “spike-in” anti-N protein antibodies. Compared to ELISA, the SH-SAW biosensor has better sensitivity and can detect anti-N protein IgG signals earlier than ELISA on day 6 (*p* < 0.05). Overall, in this study, we demonstrated that the SH-SAW biosensor is a promising platform for rapid in vitro diagnostic (IVD) testing, especially for antigen or antibody testing.

## 1. Introduction

Since the COVID-19 outbreak in late 2019, there have been more than 200 million confirmed cases (https://covid19.who.int accessed on 21 May 2021). The disease is caused by SARS-CoV-2, which is a member of the Coronaviridae family. It possesses a single-stranded and positive sense RNA genome. The genome size ranges from 26 to 32 kilobases [1,2,3,4]. SARS-CoV-2 contains four major structural proteins, including spike (S) protein, envelop (E) protein, membrane (M) protein, and nucleocapsid (N) protein [5,6]. Since the S protein and N protein are the most immunogenic proteins in SARS-CoV-2, antibodies to the S and N proteins are usually detected in serological assays. In addition, SARS-CoV-2 contains large amount of N protein. Moreover, the N protein is highly expressed during infection [7,8,9,10]. Currently, there are a number of methods to detect SARS-CoV-2, including viral culture, nucleic acid test, and immunoassay. Virus culture requires specialized laboratories and well-trained technicians. It takes a lot of time and effort to isolate the virus [11,12,13]. A common nucleic acid test is real-time polymerase chain reaction (RT-PCR). It is sensitive and accurate for the detection of viral genomes, while it requires long time and equipment. Typical immunoassays are enzyme-linked immunosorbent assay (ELISA) and lateral flow immunoassay. ELISA has the advantage of high throughput and high selectivity. However, it requires long preparation time. The advantages of lateral flow immunoassay are low cost and short time. However, some interfering factors may lead to false-negative results [14]. With the severity of the pandemic, the development of a rapid and convenient assay is important to gain insight into the duration and extent of immunization associated with SARS-CoV-2, to track the prevalence of the disease, and to assess the effectiveness of vaccines [15,16].

It has been shown that a SH-SAW biosensor can be applied to immunoassay with high selectivity and sensitivity. Furthermore, an attractive advantage of SH-SAW biosensors is the ultra-fast detection, which has been reported [17,18,19,20]. The gold membrane in the sensing area is coated with antigens or antibodies of the molecule of interest. When the assay starts, the sample is loaded onto the sensing region of the chip. The molecule of interest in the sample binds to the antigens or antibodies on the chip, and the molecular binding event causes a change in the velocity of the SH-SAW. As a result, a phase-shifted electrical signal can be obtained. The concentration of the target molecules is measured based on the detection of mass and viscosity changes in the sensing zone. When the mass and viscosity on the sensing zone increase, the velocity of SH-SAW usually decreases. In other words, the higher the concentration of the target molecule, the larger signal it produces. In addition, there are ways to amplify the output signals. Secondary antibodies or secondary antibody conjugated to gold nanoparticles can also be used for measurements. Some literature has shown that gold nanoparticles are effective in amplifying the signal and increasing the sensitivity [21].

In 2010, a research group of the authors reported a unique structure, a microfabricated air cavity and a small detection circuit for the SH-SAW biosensor chip [22]. Since the air cavity structure allows the liquid sample to reach the chip surface directly, the test procedure is very simple and suitable for point-of-care testing (POCT). We can place a drop of sample, such as whole blood, serum, urine, or saliva, directly on the chip. In addition, the air cavity structure is very efficient for the fabrication process where the capture proteins, reference proteins, and blocking proteins can be coated on the gold side of the SH-SAW biosensor. Several automated coating devices for our SH-SAW biosensor have been developed. On the other hand, since our SH-SAW biosensor does not require a washing process, a complicated flow system including pump and tubing is not required [22]. No-wash, rapid, and quantitative immunoassay SH-SAW biosensor has been expected for state-of-the-art POCT applications and several collaborative studies have been conducted over a decade [17,19,20,21,23]. These studies suggest that our SH-SAW biosensor must be one of the attractive candidates for rapid, small, and quantitative POCT kits. In this paper, since POCT must be a key technology in the recent COVID-19 pandemic, we present our SH-SAW biosensor as one of the candidates. In order to demonstrate the concept that the SH-SAW biosensor can be applied to a rapid, small, and quantitative SARS-CoV-2 antibody detection kit, we developed a SARS-CoV-2 nucleocapsid antibody detection kit for rabbits. First, we investigated the different blocking reagents and coating concentrations to explore the optimal conditions for the SH-SAW biosensor chips. Afterwards, the four parameter logistics (4PL) curve of the SH-SAW biosensor was established. The sensitivity of the SH-SAW biosensor was also evaluated. A commercially available rabbit SARS-CoV-2 nucleocapsid antibody was used as a standard. We then evaluated the production of rabbit sera collected on different days after injection of SARS-CoV-2 N protein. Secondary antibody (goat anti-rabbit IgG) was conjugated with gold nanoparticles (OD10) and used for signal amplification. For comparison with the SH-SAW biosensor, ELISA was chosen as the control experiment.

## 2. Materials and Methods

### 2.1. Materials

**Figure 1:**
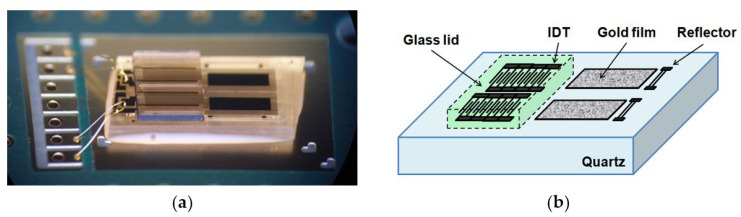
SH-SAW sensor chip. (**a**) Photograph of SH-SAW sensor chip; (**b**) schematic of SH-SAW sensor chip.

The SH-SAW sensor chips are supplied by tst biomedical electronics Co., Ltd. The SH-SAW sensor chip has a unique structure with a microfabricated air cavity above the interdigital transducer (IDT). The air cavity is composed of epoxy walls that surround the IDT and a glass lid [22]. This structure allowed the attenuation of SH-SAWs to be minimized and liquid reagents and samples can be applied directly to the chip surface. A SH-SAW sensor chip with two channels is shown in Figure 1. Each channel has three important areas: an IDT with a center frequency of 250 MHz, a sensing region with a gold film, and a reflector. The SH-SAW wavelength is around 20 μm at 250 MHz. The substrate material for the chip is 36°Y-cut 90°X-propagation quartz with a 0.35 mm thickness. The IDTs, reflectors, and sensing areas are formed with an approximately 100-nm thick gold film. The 250 MHz SH-SAW excited at the IDT propagates to the reflector, where it is reflected, and then propagates back to the IDT, where it is received [17]. This structure can reduce the size of the chip, thus reducing the cost of the chip.

**Figure 2:**
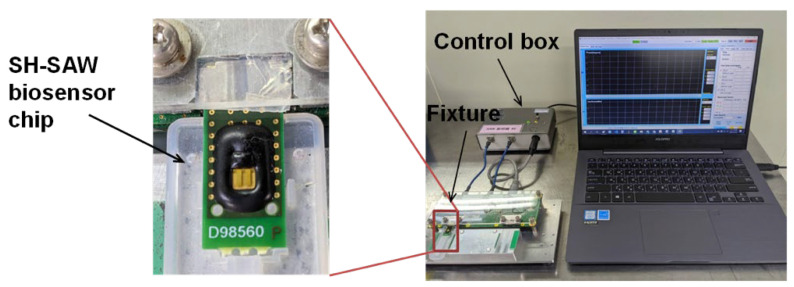
SH-SAW biosensor chip and measurement system. The left panel is an enlarged view of the measurement device in the system. The control box is connected to a fixture to which two SH-SAW biosensor chips can be connected.

In this study, we used a two-channel SH-SAW biosensor chip with approximately 3 mm × 5 mm; one channel was used for reference and the other for capture. Each channel on the chip has an IDT, a reflector, and a sensing zone between them. The chip is mounted on a printed circuit board (PCB) with the signal pads and ground pads connected by bonding wires. Afterward, the bonding wires and some areas of the chip are molded by an epoxy resin. The two-channel SH-SW biosensor chip mounted on PCB is shown in Figure 2. There is an open area of 3 mm square around the black epoxy resin, which facilitates the retention of the sample liquid. The two black rectangles in the open area are a reference channel and a capture channel with a gold film.

Hellmanex III (259304) was obtained from HellmaAnalytics. Dithiobis [succinimidyl propionate] (DSP) (VI309258) and dimethyl sulfoxide (DMSO) (TH270381) were purchased from Thermo. Recombinant N protein coated on the chips and rabbit serum were obtained from National Health Research Institutes, Taiwan. Bovine albumin serum (FUBSA001.100) was purchased from Bio Future. Stabilguard (SG01-1000) was purchased from SURMODICS. Sodium bicarbonate (BSBR8715V, Sigma) was used to dilute detection antibody when conjugating with OD10 (19120086, BBI Solutions). The serum used to dilute the standards was purchased from Sigma. Horseradish peroxidase-conjugated anti-rabbit IgG (GTX213110-01) was purchased from GeneTex. Rabbit SARS-CoV-2 nucleocapsid antibody (GTX135357) was purchased from GeneTex. The detection antibody, goat anti-rabbit (IgG) secondary antibody (ab6702) was purchased from Abcam. TMB ELISA substrate (high sensitivity) (ab171523) and 450 stop solution for TMB substrate (ab171529) were purchased from Abcam. Trizma base (SLBS16762, Sigma) with Tween 20 (0000303176, Promega) (TBST) was prepared by adding Tween 20 (0.05 v/v%) to TBS. Stabilizer were prepared by adding 15 uL of Tween 20 to 10% sucrose (BCBV9208, Sigma). Phosphate buffered saline containing Tween 20 (PBST) was prepared by adding Tween 20 (0.05 v/v%) to PBS (80 g/L NaCl, 2 g/L KH_2_PO_4_, 2 g/L KCl, 11.5 g/L Na_2_HPO_4_, pH7.4) (CWFF0613, Bio Future).

### 2.2. Fabrication of SH-SAW Biosensor Chips Coated with N Protein

Initially, the sensing area of the SH-SAW chips was cleaned with O_2_ plasma. 2% Hellmanex III was added and incubated for 20 min. After that, the chips were rinsed twice with double distilled water. Total of 0.4 mg/mL of DSP solution (in DMSO) was added onto the chips. After 20 min of incubation, the chips were rinsed with DMSO and washed with double distilled water. Then, the chips were air-dried. The reference and capture channel were coated with 2% bovine serum albumin (in double distilled water) and 1.6 mg/mL or 0.8 mg/mL of SARS-CoV-2 nucleocapsid protein (in 50 mM Tris buffer, 400 mM NaCl, and 500 mM immidazole), respectively. Afterwards, the chips were blocked with 2% gelatin (in PBS), or 2% casein pH7.4 (in PBS) to reduce non-specific binding of proteins during the detections. Finally, stabilizer was added and the chips were blown dry. The chips were labeled and stored at 4 °C.

### 2.3. Preparation of the Standard Solutions

Rabbit SARS-CoV-2 nucleocapsid antibody was diluted and used as a standard. The initial concentration of rabbit SARS-CoV-2 nucleocapsid antibody was 0.33 mg/mL. It was diluted with serum, and the final concentration was 1100 ng/mL. After that, serial dilutions were performed. The concentrations of standards were 1100 ng/mL, 550 ng/mL, 275 ng/mL, 137.5 ng/mL, 68.75 ng/mL, and 34.375 ng/mL, respectively. Serum was the blank sample.

### 2.4. Preparation of Detection Antibody Conjugated OD10

One mL of gold colloid solution was added to the Eppendorf and centrifuged at 13,000 rpm for 15 min. The supernatant was discarded. Afterwards, the pellet was resuspended with 0.05 mg/mL of the detection antibody. After incubation for 1 h at room temperature, 200 mg/mL of bovine serum albumin was added. After 30 min of incubation at room temperature, the solution was centrifuged at 13,000 rpm for 15 min. The supernatant was removed. The pellet was suspended with a micropipette and 100 µL of StabilGuard Immunoassay Stabilizer was added. The solution was then incubated at room temperature for 4 h. Afterward, the solution was centrifuged at 15,500× *g* for 15 min to remove the supernatant. Finally, 100 µL of StabilGuard Immunoassay Stabilizer was added to achieve OD10.

### 2.5. Measurement of Anti-N Protein Antibodies Using SH-SAW Biosensor

**Figure 3:**
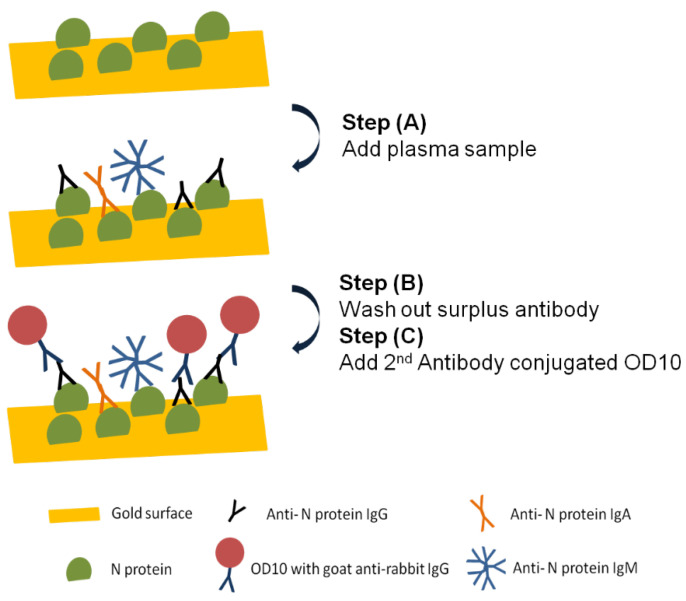
Schematic diagram of measurement protocol for anti-N protein IgG detection on N protein coated SH-SAW biosensor chip. (A) 5 µL of sample was added onto the chip coated with SARS-CoV-2 nucleocapsid proteins; (B) the chip was rinsed with PBS to remove the nonspecific-bound proteins; (C) 5 µL of detection antibody conjugated to AuNP was added.

**Figure 4:**
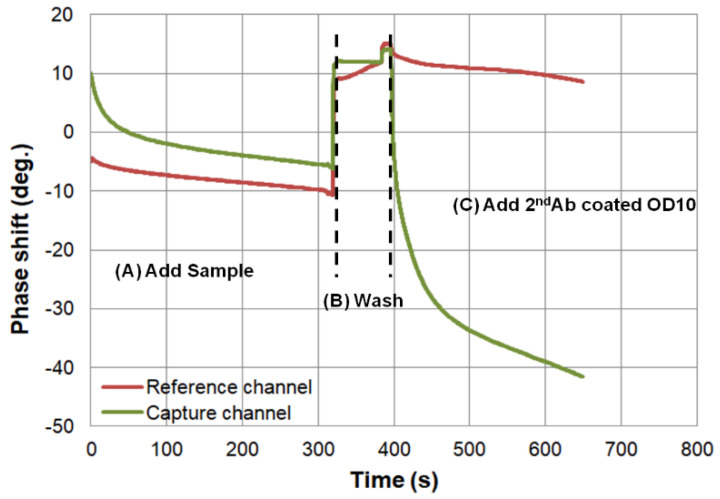
Phase shifts signals of three steps measurement with SH-SAW biosensor. The phase shifts of the SH-SAW biosensor output signals are measured continuously during assay steps (A), (B), and (C). The phase at the end of the step (B) was set as the baseline.

The SH-SAW biosensor measurement system is shown in Figure 2, which was designed by one of the research teams of the authors [22]. A control box with measurement circuitry is connected to a personal computer via a USB cable. In addition, the control box is connected to a fixture to which two SH-SAW biosensor chips can be connected. The measurement signals of the two SH-SAW biosensor chips can be displayed in real time on the personal computer and are recorded in a file. Figure 3 showed a schematic diagram of the measurement protocol for anti-N protein IgG on a SH-SAW biosensor chip coated with N-protein. In the first step (A), 5 µL of sample was added onto the chip coated with SARS-CoV-2 nucleocapsid proteins. In the second step (B), the chip was rinsed with PBS to remove the nonspecific-bound proteins. In the third step (C), 5 µL of detection antibody conjugated to AuNP was added. The molecular binding event causes a phase shift in the output signal of the SH-SAW. Figure 4 shows the phase shifts measured on the reference and capture channel during the assay, and the phase shifts of the SH-SAW biosensor output signals are measured continuously during assay steps (A), (B), and (C). The phase at the end of the step (B) was set as the baseline. The baseline and the phase at five minutes after the addition of the 2nd antibody conjugated OD10 should be related to the concentration of anti-N protein IgG in the sample.

### 2.6. Control Experiment with ELISA

ELISA was used for comparison with the SH-SAW biosensor. A total amount of 14 µg/mL SARS-CoV-2 nucleocapsid protein was added to each well of the microplate and incubated for 16 h at 4 °C. The solution was removed and each well of the microplate was washed three times with PBST. Afterwards, to prevent non-specific binding of proteins, the well was filled with 3% bovine serum albumin in PBST (blocking buffer) at room temperature. The blocking buffer was removed and each well was washed twice with PBST. Standards and samples were loaded into each well and incubated for 2 h at room temperature. The standards and samples were removed and the wells were washed three times with PBST. About 0.41 µg/mL of horseradish peroxidase-conjugated anti-rabbit IgG was added and incubated for 1 h at room temperature. After removing the detection antibody from each well, the wells were washed four times with PBST. Then, the TMB substrate solution was added to each well. After 20 min of incubation in the dark at room temperature, 450 nM of TMB substrate stop solution was added. Equal volumes of TMB substrate solution and 450 nM stop solution for TMB substrate should be used. Finally, the absorbance was measured at 450 nm using an ELISA reader.

### 2.7. Statistical Analysis

In this study, all the data were presented as means ± SD (standard deviation). Statistical analysis was performed by using Student’s t test. *p*-value less than 0.05 were considered statistically significant. Statistical analyses were performed by using SPSS statistics 17 (SPSS, Chicago, IL, USA).

## 3. Results

### 3.1. Optimization of SH-SAW Biosensor Chip Surface

To optimize the N protein coating concentration on the SH-SAW biosensor chips, we coated two different concentrations of N protein (1.6 and 0.8 mg/mL) and measured the calibration curve using the spike-in samples. The results showed 1.6 mg/mL N protein coated chips (−39.19 ± 1.35 deg.) and 0.8 mg/mL N protein coated chips (−35.64 ± 1.86 deg.). There was no significant difference between the two calibration curves. However, the 0.8 mg/mL N protein-coated chip remained one of the choices due to lower variability and cost considerations. Therefore, we chose 0.8 mg/mL N protein-coated chip.

**Figure 5:**
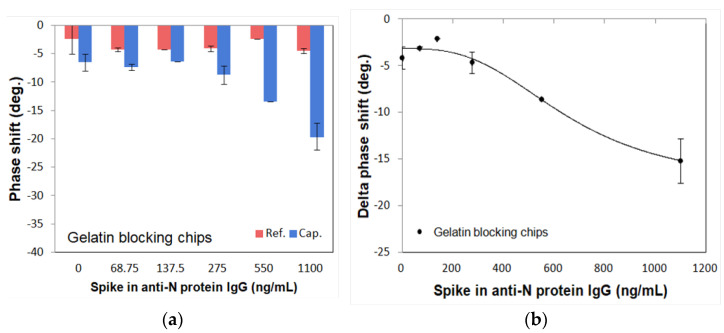
Phase shifts of gelatin blocking SH-SAW biosensor chips in different concentrations of spike-in anti-N IgG samples. (**a**) Phase shifts of reference channel and capture channel; (**b**) delta phase shifts between reference and capture channels.

**Figure 6:**
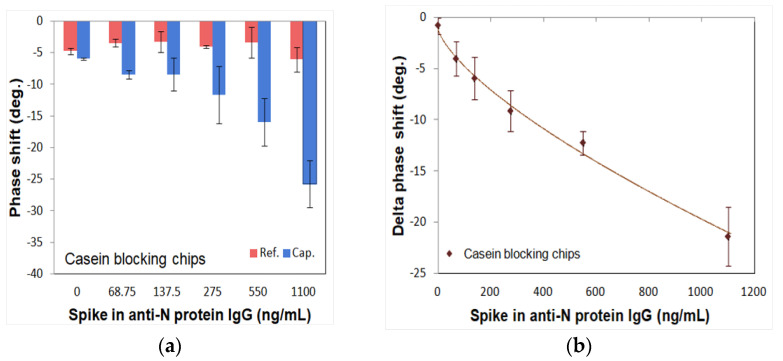
Phase shifts of casein blocking SH-SAW biosensor chips in different concentrations of spike-in anti-N IgG samples. (**a**) Phase shifts of reference channel and capture channel; (**b**) delta phase shifts between reference and capture cannels.

To optimize the blocking agents, two different blocking reagents (gelatin: 46 kDa, casein: 25 kDa) were tested using spike-in IgG samples with different concentrations from 0 ng/mL to 1100 ng/mL. Figure 5 and Figure 6 show the measured phase shifts of the reference and capture channels for gelatin and casein, respectively, as well as the delta phase shifts subtracted between them. The phase shifts of the reference channel showed almost similar values for different concentrations of N protein. On the other hand, the phase shifts of the capture channel showed different values depending on the N protein concentration. As shown in Figure 5a and Figure 6a, the phase shifts of reference and capture channels are stable and the final delta phase shifts obtained by subtracting these phase shifts are also stable for both gelatin and casein blocking reagents. In contrast, as shown in Figure 5b and Figure 6b, the 4PL correlation of the SH-SAW biosensor chips-coated N protein with the casein blocking reagent (R = 0.9711) was better than with the gelatin blocking reagent (R = 0.9511). Therefore, we chose casein as the blocking reagent.

### 3.2. Measurements of Standard Curves for SH-SAW Biosensor and ELISA

**Figure 7:**
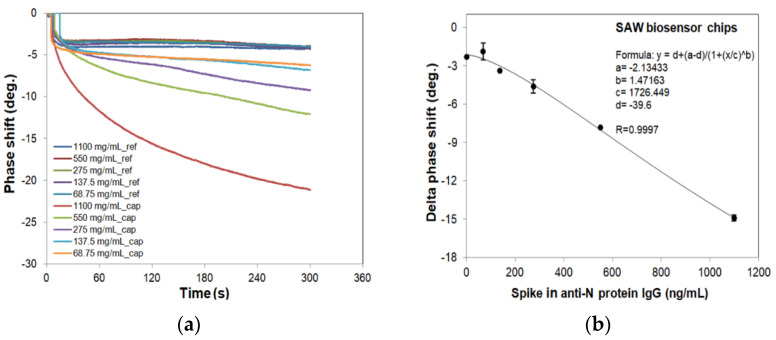
Phase shifts of SH-SAW biosensors for different concentrations of spike-in IgG samples and 4PL curve of SH-SAW biosensors. We prepared the spike-in IgG samples at different concentrations (34.375–1100 ng/mL) and blanks (serum only) for SAW chip measurements. (**a**) Real-time curve of measurement; (**b**) 4PL fitting curve.

**Figure 8:**
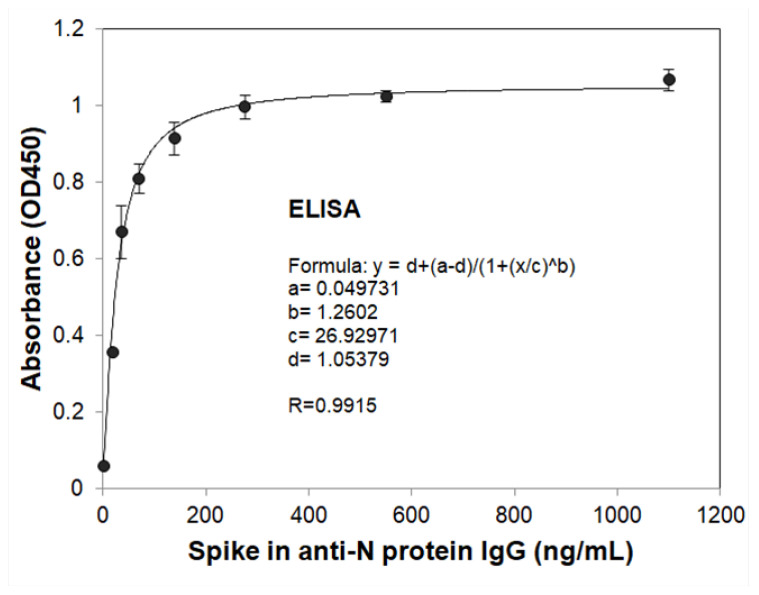
4PL curve of ELISA with OD450 absorbance signals of different concentrations of spike-in IgG samples. The spike-in IgG samples at different concentrations (34.375–1100 ng/mL) and blanks (serum only) for ELISA measurements as described in the Materials and Methods.

To evaluate and compare the sensitivity of the SH-SAW biosensor N protein chips and N protein-coated ELISA platform, the spike-in antibody samples at different concentrations (34.375–1100 ng/mL) and blanks (serum only) were measured. To create the standard curve, these phase shift signals and that of ELISA for different concentrations of anti-N protein IgG are plotted in Figure 7 and Figure 8, and fitted to the following 4PL equation:

Delta phase shift or Absorbance = D + (A–D)/{1 + ([A-N IgG]/C)^B^}, (1)

where A = −2.13433, B = 1.47163, C = 1726.449, and D = −39.6 are the coefficients for the SH-SAW biosensor chip with a coefficient of correlation (R) of 0.9997; A = 0.049731, B = 1.2602, C = 26.92971, and D = 1.05379 are for ELISA with R of 0.9915; [A-N IgG] is the concentration of spike-in anti-N protein IgG sample.

### 3.3. Measurement of SARS-CoV-2 N Protein Induced Antibodies in Rabbit Serum

**Figure 9:**
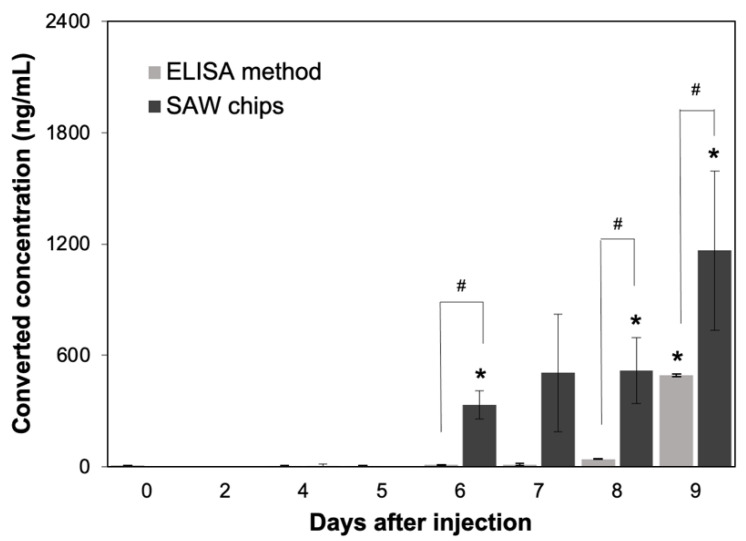
Measurement of anti-SARS-CoV-2 N protein IgG in rabbits after injection of SARS-CoV-2 N protein using SH-SAW biosensor (SAW chips) and ELISA. * *p* value < 0.05, compared with day 0. # *p* value < 0.05, which is the comparison between SH-SAW biosensor and ELISA.

We used the SH-SAW biosensor and ELISA to measure the anti-N specific antibodies produced by rabbit sera collected at different days after SARS-CoV-2 N protein injection. From the results, we observed a surge of rabbit serum IgG in rabbit on day 6 of N protein injection. We compared the SH-SAW biosensor and ELISA by measuring N protein-infected rabbit sera Figure 9, and the results showed that SH-SAW biosensor could detect rabbit serum IgG on day 6 with a significant phase shift change (*p* value < 0.05); however, ELISA could only detect rabbit serum IgG with a significant signal change (*p* value < 0.05) on day 9.

## 4. Discussion

From the outbreak of the COVID-19 epidemic in 2020 to the present, the number of coronavirus cases has exceeded 219 million. There is an urgent need for a rapid and accurate coronavirus detection method [24,25,26]. The objective of this study was to develop a novel SH-SAW-based biosensor coated with SARS-CoV-2 N protein and measure SARS-CoV-2 antibody in rabbit serum using SAW technology.

SH-SAW technology was designed and built by the IDT design to generate acoustic waves on a solid surface, allowing us to directly observe changes in the wave characteristics of a specific marker when it binds to the SH-SAW surface. Compared to ELISA methods, SH-SAW technology is easier and faster to obtain real-time results because there are no secondary antibodies or signal enhancers. Other technologies, such as surface plasmon resonance (SPR) technology, can also achieve high reliability, sensitivity and repeat-free measurement by measuring the variation of the sensing angle over time; however, the SPR detector systems are larger and more expensive than SH-SAW detection systems [27,28]. Electrochemical methods can also provide label-free measurements by measuring the change in current or potential as the biomarker binds to the surface, but the principle of electrochemical methods involves oxidation-reduction reactions, which may affect the stability of the biosensor [29,30].

To construct a simulation of COVID-19 infection, we injected the rabbits with recombinant N protein and measured anti-N protein IgG in rabbit serum after different days of injection by SH-SAW biosensor. We successfully developed a construction scheme for a prototype SH-SAW biosensor for measuring anti-N protein IgG in serum. The reference channel of the dual-channel SH-SAW biosensor chip reduces environmental or non-specific effects such as changes in temperature and sample viscosity. By using a miniature dispensing machine to load the coating materials onto the gold films in the sensing and reference zones of the chip, the machine allows accurate and precise control of liquid volume up to 40 nL [31,32]. N protein was cross-linked to the gold film of the chips via disulfide bonds, and DSP has been widely used for the attachment of gold nanoparticles. In order to obtain better resolution of SH-SAW signal, the blocking agent is one of the key factors to optimize the SH-SAW phase shift signals. If the molecular size of blocking agent is too large relative to the coating material, the coating material will be obscured by the blocking agent, making it difficult to capture its target. From the results, the chips blocked with casein as the blocking agent to perform a better phase shift signal because of the smaller shielding effect. Although many ELISA COVID-19 antibody assays are now available in the market, the detection time of ELISA is not convenient for large-scale screening [33,34,35]. The SH-SAW biosensor has an advantage of real-time measurements, which takes only about 10 min per measurement, whereas it takes at least 6 h to perform ELISA. In addition, the SH-SAW biosensor is portable. Measurements can be performed anywhere [20]. Measurements with the SH-SAW biosensor are reproducible. It can be used for semi-continuous measurements [17]. The sample volume used in the SH-SAW biosensor measurements is very small. Only 5 μL of sample is required for the measurement, which is another advantage of SAW biosensor technology compared to ELISA. In addition, we used SH-SAW biosensors and ELISA to measure rabbit sera that were obtained from different days after N protein injection. The results showed that SH-SAW biosensor has better sensitivity and larger signal than ELISA.

Despite the many advantages of the SH-SAW sensor, some aspects of the current SH-SAW biosensor limit the usefulness of this device. The SH-SAW biosensor is measured in a three-step process; the more measurement steps there are, the greater the potential impact on the accuracy of each test. However, this can be demonstrated by pre-operational training to familiarize practitioners with biosensor measurements or by mixing samples directly with lyophilized OD10 enhancer, omitting the cleaning step, thus enabling one-step sample addition. The SH-SAW biosensor is a novel technology for in vitro diagnosis devices (IVD) and POCT, and the extremely short test time is its greatest advantage which is worth of development in more aspects of biomarker IVD. In addition, the measurement data as shown in Figure 7a indicate molecular binding events on the surface. The binding kinetic analysis can help to further improve the measurement accuracy, in addition to monitoring the sensor output changes. Study is ongoing on the binding curves of the SH-SAW sensor system [36,37].

## 5. Conclusions

Due to the COVID-19 pandemic, there is an urgent need for a rapid and convenient point-of-care testing product. The SH-SAW biosensor platform coated with SARS-CoV-2 N protein provides a highly sensitive and rapid anti-N protein IgG test. Although the operational steps and OD10 stability have yet to be optimized, the SH-SAW biosensor platform has the potential to be developed for more aspects of biomarker IVD.

## Data Availability

The data set generated and/or analyzed during the study can be requested from the corresponding author.
